# On the Epidemic Distempers of the Year 1802

**Published:** 1804-04-01

**Authors:** Nathaniel Potter

**Affiliations:** Baltimore


### *On the* Epidemic Distempers *of the Year* 1802. *By* Nathaniel Potter, *M. D. of Baltimore. Communicated in a Letter dated January* 28, 1803.

### MEASLES.

THE year 1802 constitutes a very interesting crisis in the history of American epidemics, more especially to those who view the Measles, Scarlatina, and Yellow Fever, as originating from one great general cause, and, consequently, as links of the same pestilential chain.

The winter 1801-2 was the dryest and warmest that can be remembered by the oldest inhabitants of our city. The mercury stood above the freezing point not only during the day, but through the night, much the greater part of the winter. The gutters, docks, and other repositories of filth, emitted an effluvium little less offensive than that which is exhaled during the summer and autumnal months. Whether the result of putrefaction contributed in any degree to modify the characters of our winter epidemics, I shall not pretend to decide; but state the most prominent symptoms and greatest variations that occurred to my own observation, and leave the learned to deduce such conclusions as they may think reasonable.

Agreeably to the report of some of our practitioners, the measles commenced their reign about the first of January; but the symptoms were not completely developed in any case that occurred to my observation until the 15th of that month. During the months of November and December, I had remarked the unusual frequency of an indistinct efflorescence, which was occasionally associated with every assemblage of symptoms which might have been occasion-

 ed ed by the vicissitudes of the weather. It appeared to me to be the evanescent shade of the scarlatina retiring before the majesty of a more terrible enemy. It is true that the scarlitina had not been epidemic, but decided cases had occurred sporadically, not only in November and December, but during all the last year. This efflorescence was associated with inflammations of the parotid glands, and of the tonsils, with inflammation of the lungs and pleura, and with symptoms of catarrh, intermittent fever, and dysentery, and with a variety of anomalies that have no name in nosology, until it was suffocated with them all by the arbitrary interposition of the epidemic measles. No sooner had this disease discovered its proper features than every other fled before it; and what the poet says of Fame, may be justly applied to this as well as to other great epidemics;

"Mobilitate viget viresque acquirit eundo."

So truly despotic were the causes producing measles, that even many of those who had suffered their influence in former epidemics were not entirely exempted. This miniature picture of the genuine disease appeared in several forms, viz. in catarrhal symptoms, in slight inflammations of the throat, in a sense of heaviness over the forehead, in a frequent watery discharge from the eyes, and, in a few cases, it was manifested by an eruption and a morbillous cough. These affections gave rise to an opinion that the system could be susceptible of measles more than once, and I shall say hereafter that this opinion was revived in spring, when the scarlatina arrogated the same dominion over the measles that the measles had done over the minor diseases of the winter. This second sensibility to the causes of measles seemed to be heightened by some inviting predisposition; but, above all, by previous diseases of the lungs. Adults were much more susceptible than children to this second impression; whence it would seem that the latter laboured under fewer predispositions, or that the former had partially lost the former morbillous impression. I did not see a person under ten years old affected with the slightest symptom, who had the disease when it was epidemic here six years ago. I saw no vestige of it in subjects above forty years old. Children were less violently affected by genuine measles than adults, but there were few grown persons who had not previously experienced them. This epidemic did not appear to me to merit the appellation of contagious in the same degree as small-pox; nor, indeed, in a very considerable degree. The causes seemed to to me rather derived from the atmosphere than from a sick person; for many persons resided in families where the disease prevailed, and even slept with measly children with impunity. Should it be contended that measles are, strictly speaking, communicable from a sick to a well person, and should the opinion be well founded, is it not probable that some morbid or other strong impression had previously rendered such persons unsusceptible of impression from morbillous effluvia, just as the vaccine disqualifies the system for the reception of impression from variolous matter? There are, no doubt, many agents yet to be discovered that may act as eternal preventives of the most deleterious poisons. Will he not be entitled to immortality who shall, by such a discovery, obviate the effects of the saliva of a mad dog, or of the plague-engendering poison?

The symptoms of measles are so well known that I shall only notice such as do not constantly occur, or such as require attention in a therapeutic point of view. As in other epidemics, so in this, there were many cases so mild as not to require the interference of a physician: indeed, it was so light in some children that none but the physician discovered it. The head was more violently affected with pain and tension over the forehead than is common in the measles, although the swelling of the face was less considerable. The affection of the breast was the most important consideration, as the greatest danger was to be apprehended from effusions in the lungs: indeed, the organs of respiration, and parts adjacent, were affected in all the intermediate degrees between a slight cough and a most inflammatory fever. In some children the trachea and bronchial vessels where so highly excited as to occasion symptoms of croup. The affection of the stomach did not amount to that degree of puking which is so often mentioned by writers on measles; but a deadly sickness and a corroding sensation were constantly complained of. The affection of the stomach was less violent in adults than in children. The diarrhœa, so generally noticed in books, was seldom a symptom of this disease; never, that I know of, unless in consequence of neglected evacuations. The danger of the disease was in no wise in the ratio of the violence of the attack; for the cases that often proved most formidable manifested no turbulent symptoms until about the seventh day, when a sudden pulmonic affection often prostrated the system in a few hours. The ear-ach was often a symptom in children, as well during the inflammatory state as during convalescence. The eyes were sometimes highly inflamed, although ophthalmia was oftener a consequence than a symptom of the disease.

The cure of the measles this year may be almost reduced to two simple remedies, *blood-letting* and *purging*; for when these were used in time, and carried to a sufficient extent, little or nothing remained to be done. These remedies were no less efficacious in removing the immediate symptoms than in preventing the consequences of the disease. This will be sufficiently apparent when we enumerate the deplorable train of consequences that followed their neglect. Such was the inflammatory grade of this disease, that a repetition of the use of the lancet was frequently required to the fourth or fifth time. As long as any symptom indicated the smallest relict of inflammation, the lancet was the most safe and efficacious remedy. The pulse required much attention, in some cases, to perceive the necessity of this remedy. Excessive inflammation or infarctions of the lungs, bronchia, or trachea, often obstructed the process of respiration in such a degree as to influence the pulse very materially. The skin was often cold, the cough laborious and unproductive, with all the symptoms of great indirect debility, with a pulse scarcely to be felt, and yet no remedy but venæsection could be depended on for relief; for the pulse rose after it, and free distinct action developed itself in all cases where the prostration had not continued a considerable time. This affection of the breast constituted the principal danger in this disease, but never produced death where the lancet was used early, and carried to a proper extent. Where the system had remained long in this semi-suffocated state, the lips and nails appeared blue, and a dark grumous blood was often expectorated. These were commonly the gloomy presages of approaching dissolution, unless the heart and arteries reacted after bleeding, which was generally the case when it was practised before the ninth day, and sometimes at a more advanced state of the disease. In this disease, as in all others where indirect debility is induced by excess of arterial action, bleeding, to be useful, should be practised with great caution. Large bleedings can never be used with propriety where this state has long existed, and the longer it has existed the less should be the quantity.

The blood drawn from a vein was uncommonly florid when drawn while arterial action was vigorous and the pulse frequent, but lost its floridity as soon as the functions of the lungs became impaired, or indirect debility supervened. In the different degrees of prostration it manifested ed the following different appearances. 1. A copious deposition of a red precipitate to the bottom of the receiving vessel: this was deemed to be the first indication of a tendency to dissolution in the blood. 2. Blood of a darker complexion, separating imperfectly, with the serum bloody, and the crassamentum like blood half coagulated. 3. A total dissolution of the whole mass, appearing like molasses or tar, without a separation, and very black. Some have imagined that this tendency to dissolution is occasioned by the miasmata acting as the cause of the measles; but such an hypothesis seems improbable, as it occurs in pleurisy, and in many diseases of excessive, long-continued inflammatory action; though it must be confessed, that it occurs very often in such diseases as are called contagious. That the blood should be completely dissolved, and in a few hours after manifest a buffy coat, is almost an unanswerable argument against the solvent power of the miasma. This I have witnessed in measles, small-pox, pleurisy, yellow fever, and in other diseases. Does not this fact carry with it a strong presumption against the vitality of the blood? In the dissolved state it cannot be supposed to possess life, and it is equally difficult to suppose that it could recover its vitality in a few hours; nor is it probable that new blood could be so suddenly generated, when the chylopoetic functions are almost, if not altogether suspended. The blood was seldom sizy in this disease: the grade of the epidemic was so malignant that this appearance was seen only in mild cases.

Purging, though not as essential to the cure as blood-letting, was, nevertheless, a very useful remedy: calomel acted like a charm in removing the corroding nauseous sensation at the stomach. It was more grateful to that organ than any other purge, and required to be repeated every second day, or oftner, as there was a constant re-accumulation of that green acrid matter that was sometimes ejected from the stomach on the first attack; and this disposition commonly lasted four or five days. Where purging was neglected in the commencement, the evacuations from the intestines were often of a dark green, brown, or black complexion, just as it happens in other malignant fevers.

Emetics were sometimes useful in removing excreted mucus from the bronchia and trachea, though they could not be used with propriety in the early or inflammatory state, because they increased the inflammatory action, and particularly the determination to the head. They often afforded a temporary relief of the nausea at the expence of increasing creasing the fever. They proved more useful in the advanced state of the measles, when indirect debility began to progress, provided no decided local affection forbade them.

Antimonials were certainly improper medicines in this disease, they depressed the pulse, and seemed to act too much like the causes of the disease. Are not antimonials equally unfit remedies in all malignant fevers where the tendency to indirect debility is great, and more especially in those called contagious, where the *vis nocens* is so prone to induce the same state of the system?

Blisters were equally inapplicable in the first state of this disease, but co-operate powerfully with emetics in arresting the progress of indirect debility in the advanced state of measles, and sometimes called forth dormant excitement to great advantage.

Opium was equally inadmissible in all its forms, unless towards the latter state, when fever did not contra-indicate its prescription for the cough, which was often the last troublesome symptom, and seemingly occasioned by the action of a small portion of the pulmonary vessels.

Although measles generally run their course, in all cases, nearly in the same number of days, they manifested a trait discoverable in all malignant fevers; viz. a disposition to terminate in a shorter space immediately before they disappeared. This signal for the cessation of hostilities is very remarkable in scarlatina, yellow fever, dysentery, jail-fever, and others. The action of the morbillous fever produced one effect which I never saw from any other morbid action: it increased all the symptoms of gonorrhœa, and fomented that local action to a degree unknown to me. It was moreover evident, that, from the commencement of the measles to the termination of our autumnal epidemic, that disease not only resisted the common remedies, but yielded, in many cases, only to all the force of the whole train of antiphlogistic remedies. The difficulties attending the treatment of this disease, formed, during that period, a subject of astonishment and complaint for the practitioners of our city.

I should not have been thus particular in detailing the phenomena of measles, were it not that the popular opinion supposes them to be void of danger, and, therefore, seldom requiring the interference of a physician. A more hazardous notion cannot exist; for the neglect of no disease originates more deplorable consequences, even where the patient recovers. It therefore appears to me that this constitutes constitutes the most important part of the subject, I shall enumerate some of the most common consequences of neglected or maltreated measles; although the limits of a letter must abridge the list considerably.

1. Effusions in the lungs. 2. Effusions in the brain, constituting what are called cases of hydrocephalus internus; these soon produce death. 3. Debility of the lungs, inviting disease by constant predisposition. 4. Cough, often terminating in consumption, as well as the state immediately preceding. 5. Slow convalescence by the tedious process of expectoration. 6. Inflammations of the eyes were common; the palpebræ were highly inflamed both during the disease and long after its decline, and the cornea was often so completely covered with an excrescence as to obstruct vision partially or entirely; this was both a symptom and consequence of measles. This last symptom occurred principally among children, and began about the second or third day. 7. As ear-ach was often a symptom, so internal ulceration was a consequence of measles; and these frequently proved very difficult to cure. These occurred mostly in patients under puberty; and I met with several cases that resisted all the ordinary remedies, and yielded only to a salivation. Some were cured by injecting weak solutions of corrosive sublimate into the ulcerated ears. 8. Eruptions on the skin, comprehending several anomalies, but consisting more frequently of a thickening of the cuticle, similar to the affection of the cornea, which may be justly viewed as the scarf-skin of the organ of vision.—These probably make no figure as to the whole of the evils resulting from neglected or ill treated measles; but they are sufficient to deter all reasonable men from trusting to the wild aberrations of nature in the cure of so dangerous a disease; and it is certainly a very consolatory reflection, that none of them followed measles where *bleeding* and *purging* were judiciously used.

Before I relinquish this subject, I must be permitted to describe one case of measles; to omit it would be to lose a pathological curiosity, the parallel to which (so far as I know) is not to be found in the annals of medicine.

On the 15th of February I visited Ann Watson, aged eleven years, who had been attacked with the usual symptoms of measles five days before. She had been a healthy child from her infancy; and the immediate cause of calling for medical assistance was an affection of the throat unusually violent for this disease. Excepting this symptom, nothing uncommon was discovered until the eighth

 day, day, when a coarse furfuraceous scale was discovered on each point where the eruption had originally appeared; from these this thickening of the cuticle diverged, and in three days it enveloped the whole surface of the body in one universal scale. This induration of the cuticle increased daily, and, by the fifteenth, it had acquired the hardness and thickness of the nails, and apparently in no respect different from them. This scale was not confined entirely to the external surface of the skin; it was perceptible (though in a smaller degree) on the insides of the ears, nose and mouth, and as far down the œsophagus as could be seen. Previous to the eruption, as soon as the cough began to agitate the lungs, an unusual degree of redness instantaneously diffused itself over the face; not like the hectic blush (so common in inflammatory affections of the lungs,) confined to the cheeks, but extending over all the face. So violent was the influence of the lungs upon the skin, that this scarlet efflorescence, in some long paroxysms of coughing, extended over the whole body. The hair was materially interested in this symptom. The same substance was generated, in great abundance, from its roots, and seemed to be occasioned by a superfluous secretion of the substance forming the hair. Every hair acquired, at its root, the thickness of many hairs, and a whiteness, strongly resembling the *Plica Polonica**, as described in books. Such was the thickness and hardness of this horny excrescence, that scales of it were frequently pared off without the least sensation. For some days before death, the least motion of the body produced extreme pain; and, when she walked, a sound was emitted from the body as if she had been cloathed in buckram. On the twelfth day she became considerably blind and deaf. The cornea thickened until it put on the appearance of having been burnt, and total loss of vision ensued. The deafness did not progress in the same ratio, as the hearing continued, in a small degree, as long as she lived. The sense of smelling was also considerably impaired. The sense of tasting was entirely lost. Sugar and salt, and even brandy and milk, had the like effect on the tongue and fauces. The understanding was not impaired, nor the least symptom of derangement discoverable through the whole disease. From the commencement

* Is this disease, so common in Poland, an increased secretion and deposition of the same nature?

mencement of the induration of the cuticle, the symptoms became more and more aggravated. The cough was frequent, hard, and unproductive, but little pain was felt in any part of the thorax, and no expectoration appeared to the last. The greatest pain was experienced in exercising the muscles of deglutition, and the thought of attempting it frequently occasioned the most violent convulsions, in one of which she expired while attempting to drink, on the nineteenth day from the attack. Every idea, however remotely connected with the act of swallowing, occasioned the greatest trepidation of mind, and agitation of the whole nervous system; in short, these symptoms recalled to my mind all the horrible scenes of a most deplorable case of hydrophobia which I witnessed at eleven years of age. No pain of the head was complained of from the beginning. The most excruciating pains through the whole alimentary canal were felt, though not constantly. On my first visit she was bled, and a temporary relief from the cough was obtained. The blood was uncommonly sizy. Every attempt to evacuate the intestines completely proved abortive, although the attempt was made daily, for fifteen days, with the most powerful cathartics. The warm bath was resorted to in the violence of pain; but the relief produced by immersion, however long continued, was scarcely perceptible. Opium, for the four or five first days, produced a temporary relief, but at length it effected nothing more than the same quantity of water. With a view of relaxing the rigid cuticle, the body was covered with warm olive oil, with the longest respite from pain that she experienced; and large doses internally, afforded considerable alleviation of the tormina of the intestines. I regret that I was not permitted to examine, by dissection, the viscera and other internal parts, and must, therefore, leave the subject for the contemplation of the learned.

